# Lower AKI Risk and Higher Survival With Microaxial Flow Pump Support During Elective PCI: A Propensity Score‐Matched Study

**DOI:** 10.1002/ccd.70721

**Published:** 2026-07-06

**Authors:** Elric Zweck, Andrew Shaw, Amin Polzin, Nudrat Noor, Sathya Unudurthi, Michael Cuchiara, Kai M. Schmidt‐Ott, Malte Kelm, Ralf Westenfeld, Rafael Kramann, Ravindra L. Mehta

**Affiliations:** ^1^ Department of Cardiology, Pulmonology, and Vascular Medicine, Medical Faculty Heinrich Heine University Düsseldorf Düsseldorf Germany; ^2^ Abiomed, Johnson & Johnson MedTech Danvers Massachusetts USA; ^3^ Department of Nephrology and Hypertension Hannover Medical School Hannover Germany; ^4^ Department of Medicine 2 (Nephrology, Rheumatology, Immunology), Medical Faculty RWTH Aachen University Aachen Germany; ^5^ Department of Internal Medicine, Nephrology and Transplantation Erasmus Medical Center Rotterdam the Netherlands; ^6^ Department of Medicine University of California San Diego San Diego California USA

**Keywords:** acute kidney injury, interventional cardiology, kidney outcomes, mechanical circulatory support, renal replacement therapy

## Abstract

**Background:**

Acute kidney injury (AKI) is associated with worse outcomes after percutaneous coronary intervention (PCI). Previous, smaller studies suggested nephroprotective effects of micro‐axial flow pump (mAFP) during PCI. This study aimed to compare renal outcomes of high‐risk PCI with mAFP or intraaortic balloon pump (IABP) support.

**Aims:**

This study aimed to compare renal outcomes of high‐risk PCI with mAFP or intraaortic balloon pump (IABP) support.

**Methods:**

Patients undergoing elective, non‐emergent, high‐risk PCI with mAFP or IABP between 2017 and 2025 were retrospectively included from the TRUVETA database, which covers 120 million US patients. Patients with mAFP or IABP support were propensity score‐matched for AKI risk, adjusting for demographic and clinical characteristics including baseline kidney function, AKI risk, and comorbidities.

**Results:**

Of 24,645 patients undergoing PCI with mechanical circulatory support, 7872 elective high‐risk PCI cases were identified: 2349 (29.8%) with IABP and 5523 (70.2%) with mAFP support. Prior to matching, patients receiving mAFP support were older and had more comorbidities compared to IABP. After exclusion of patients with missing baseline eGFR, propensity score matching was performed in 4666 patients (2333 per group) resulting in comparable baseline characteristics of the two groups. After matching, the risk of AKI (18.9% vs. 23.2%) and all‐cause mortality (15.9% vs. 20.7%) was lower in mAFP‐supported patients compared to IABP (all *p* < 0.001). Bleeding, stroke, and RRT incidences were comparable between groups. There was no interaction between baseline kidney function and lower AKI risk with mAFP.

**Conclusion:**

This propensity score‐matched observational study indicates that the use of mAFP in the setting of elective high‐risk PCI is associated with a lower risk of AKI and higher survival compared with IABP.

**Abbreviations**
AKI: acute kidney injury
eGFR: estimated glomerular filtration rate
IABP: intra‐aortic balloon pump
mAFP: microaxial flow pump
PCI: percutaneous coronary intervention

## 1 Background

As percutaneous coronary intervention (PCI) has become a cornerstone for the treatment of coronary artery disease, advances in interventional techniques and devices have expanded the indications for PCI to include patients previously considered ineligible for revascularization due to complex coronary anatomy, severe comorbidities, or hemodynamic instability. In these high‐risk patients, mechanical circulatory support (MCS) devices, such as micro‐axial flow pumps (mAFP) like the Impella CP and intra‐aortic balloon pumps (IABP), have emerged as adjunctive therapies to provide hemodynamic support during PCI, with the goal of stabilizing hemodynamics, reducing ischemic burden, and facilitating complete revascularization [1, 2].

Acute kidney injury (AKI) after PCI is associated with increased morbidity and mortality, prolonged hospital stay, and a higher likelihood of long‐term renal dysfunction [3]. The mechanisms contributing to AKI in these patients are multifactorial and include contrast‐induced nephropathy, hemodynamic fluctuations, and inflammation [4, 5]. The effects of MCS device utilization on the risk of renal complications are unclear. In high risk PCI patients, mAFP use has demonstrated advantages in procedural feasibility, short‐term outcomes, and retrospective analyses suggest lower rates of renal injury after PCI associated with mAFP utilization [6, 7]. Conversely, in acute infarct‐related cardiogenic shock patients (DanGer Shock Trial), mAFP use was associated with increased rates of renal replacement therapy (RRT) [8, 9]. However, the DanGer Shock trial included different patients in a setting drastically different from elective high‐risk PCI confounding this results applicability to PCI [10]. Therefore, further evidence is needed to better understand the association of MCS on renal outcomes in elective high‐risk PCI.

Novel large data platforms, utilizing real‐world data (RWD) such as electronic health records and diagnosis code‐based databases, offer potential advantages over prospectively enrolled clinical trials such as greater generalizability, improved efficiency (cost and time), and larger data sets enabling observational studies that help bridge the gap between prospective controlled clinical trials and clinical practice.

The objectives of this analysis were to describe the characteristics of patients undergoing elective high‐risk PCI procedures, supported by either mAFP or IABP and to compare the risk of AKI, RRT, and all‐cause mortality in high‐risk PCI patients supported by mAFP versus IABP, using large‐scale RWD accounting for available confounders.

## 2 Methods

### 2.1 Data Source

This retrospective, observational study utilized data from the Truveta (Truveta Data, Bellevue, WA, USA) Electronic Health Record (EHR) Database, covering the period from January 1, 2017, to October 27, 2025. The Truveta EHR Database aggregates de‐identified electronic health record data from a diverse network of 30 healthcare systems across the United States covering a total of 120 million patients that represents 18% of clinical care in the United States (Truveta, Bellevue, WA, USA). It provides comprehensive real‐world clinical information, including patient demographics, diagnoses, procedures, laboratory results, medication usage, and clinical outcomes. Truveta's data undergoes rigorous quality control processes to ensure accuracy and consistency, making it a reliable source for large‐scale observational studies. The database enables longitudinal tracking of patients, allowing for the assessment of outcomes over time and the identification of trends in clinical practice.

Patient demographics, including date of birth, sex, and race, were extracted from the EHR data at the point of admission.

Medical conditions and comorbidities were identified using ICD‐10 diagnosis codes, procedural events were defined using current procedural terminology (CPT) codes, and laboratory results were classified based on Logical Observation Identifiers Names and Codes (LOINC) [11].

### 2.2 Study Population

For the present analysis, the initial patient population included individuals who underwent high‐risk PCI procedures supported by either mAFP (Impella CP, Abiomed Inc., a subsidiary of Johnson & Johnson MedTech, Danver, MA, USA) or IABP. For each patient, the most recent PCI was selected, if multiple were recorded. Subsequently, the following exclusion criteria were applied: extracorporeal membrane oxygenation therapy, multiple MCS devices applied within the same day of PCI, emergent admission, cardiogenic shock, ST‐elevation acute myocardial infarction, right heart failure, subsequent coronary artery bypass graft surgery after the procedure and—for the clinical outcome analyses only—missing pre‐PCI creatinine values, that is, missing baseline estimated glomerular filtration rate (eGFR). Exclusion criteria were identified using ICD‐10 codes.

### 2.3 Outcome Assessment

The primary outcomes of interest for this study were the need for RRT, all‐cause mortality, and the occurrence of medical code‐derived acute kidney injury (cd‐AKI) within 90 days after PCI. Safety outcomes, stroke, and bleeding requiring transfusion, were also assessed 30 and 90 days after PCI. For the timing of events, we used the dates that the event was reported first with any of the codes after the index date.

All‐cause mortality was assessed utilizing data from a third‐party vendor (LexisNexis, New York City, NY, USA) that retrieved this information via social security information. This data source was chosen over hospital codes for mortality assuming higher reliability and completeness, as well as independence from hospital systems.

cd‐AKI was defined as the occurrence of any AKI ICD‐10 diagnosis codes if not present already at admission (see Supporting Information S1: Table S7 for codes used). Due to the lack of serial creatinine measurements in the database for the majority of patients (< 20% of patients with baseline creatinine had creatinine labs collected within 14 days of procedure), no creatinine lab values were included in the definition of AKI.

RRT was defined as the occurrence of any RRT CPT code or ICD 10 diagnosis code (see Supporting Information S1: Table S7 for codes used).

Stroke and bleeding requiring transfusion were defined by ICD diagnosis codes (see Supporting Information S1: Table S7 for codes used).

### 2.4 Statistical Analyses

We first examined characteristics of the patients in both the IABP and the mAFP group prior to matching, focusing on demographics, past medical history, laboratory parameters, and basic hemodynamics. Then, propensity scores were calculated using simple logistic regression based on pre‐selected potential confounding covariates which encompassed age, sex, history of anemia, history of heart failure with reduced ejection fraction or history of chronic heart failure, history of insulin‐dependent diabetes, index visit non‐ST‐elevation myocardial infarction (NSTEMI), admission eGFR (2021 Chronic Kidney Disease Epidemiology Collaboration [CKD‐EPI] creatinine equation) [12], and variables predicting bleeding risk (history of transfusion due to bleeding, history of liver disease, history of cancer). Importantly, these selected confounders also included known predictors of AKI risk, as represented by their appearance in the Mehran score, a validated and frequently used metric in PCI patients [13]. All variables used for matching were pre‐procedural to avoid collider stratification bias [14]. All these variables were available in the final study population, avoiding the need to impute missing values. Propensity score matching was carried out using nearest‐neighbor 1:1 matching without replacement utilizing a caliper width of 0.1 standard deviations of the propensity scores to determine matching eligibility. Absolute standardized mean differences were calculated pre‐ and post‐matching for all selected covariates. The propensity scores pre‐ and post‐matching were plotted in histograms to visualize matching effectiveness. We reassessed the aforementioned patient characteristics in the IABP and the mAFP group after matching. The risk of AKI was assessed in both groups separately and also stratified by chronic kidney disease stage as determined by admission eGFR. We then proceeded to compare the prespecified 30‐ and 90‐day outcomes between the matched patients in both groups. To address potential survivorship bias, renal outcomes were also calculated and reported separately among 30‐ and 90‐day survivors. Finally, to assess the relevance of the renal endpoints for mortality, in the matched cohort, across both groups and within each group, we calculated the odds of 90‐day mortality among those with specific renal events compared to those without these renal events. Data are presented as means with standard deviations or counts with proportions. Comparisons between groups were performed using *t*‐tests for continuous variables and chi‐square tests for categorical variables. Univariate logistic regression models were used to calculate odds ratios for the prespecified outcomes in the mAFP versus the IABP group. To explore the effects of baseline kidney function on the renal outcomes, we stratified the matched cohorts into baseline eGFR categories. eGFR was calculated using the CKD‐EPI 2021 formula [12]. The risk of AKI was then calculated for each eGFR category and odds ratios were calculated for each category, compared to eGFR above 60 mL/min as the reference. We also built logistic regression models with AKI as the outcome and with an interaction term between eGFR category and the IABP/mAFP group assignment to explore whether baseline kidney function contributed to outcome differences between the groups. Finally, to address potential survivorship bias, renal outcomes were also calculated and reported separately for 90‐day survivors in both groups. An *⍺* level of 0.05 was considered statistically significant.

### 2.5 Ethical Statement

We first examined this observational cohort study, which used only de‐identified patient records obtained from the Truveta EHR platform and did not require institutional review board approval or informed consent. De‐identification was verified through expert determination consistent with the Health Insurance Portability and Accountability Act Privacy Rule. The study was conducted in accordance with the principles of the Declaration of Helsinki.

## 3 Results

### 3.1 Patient Characteristics Before and After Propensity Score Matching

**Figure 1:**
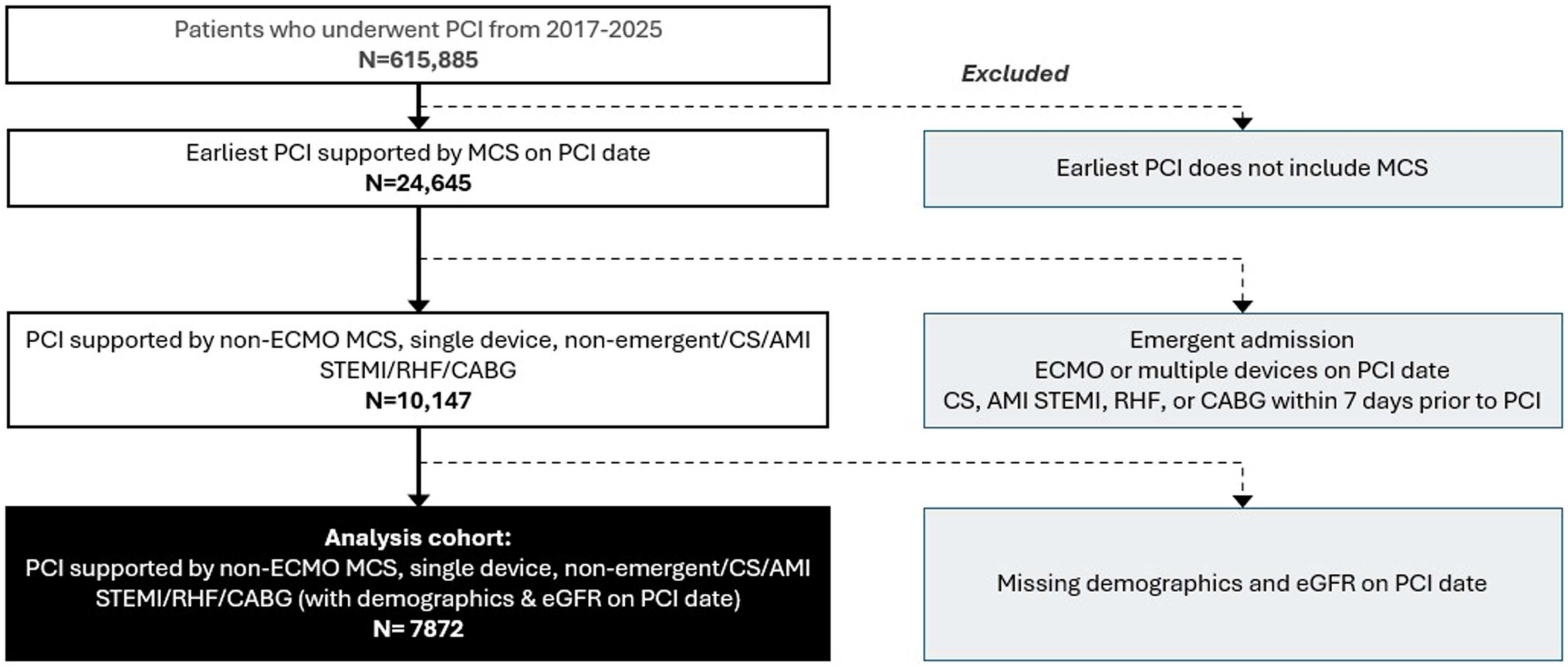
Study consort diagram. Subjects without MCS, ECMO, multiple MCS devices on PCI date, subjects with an emergent admission or comorbidities were excluded. Subjects with missing demographic data or eGFR on PCI date were excluded. AMI STEMI, acute myocardial infarction ST segment elevation; CABG, coronary artery bypass graft; CS, cardiogenic shock; ECMO, extracorporeal membrane oxygenation; MCS, mechanical circulatory support; PCI, percutaneous coronary intervention; RHF, right heart failure. [Color figure can be viewed at wileyonlinelibrary.com]

Of the 615,885 PCI patients in the TRUVETA database during the respective period, 24,645 underwent PCI with MCS. Out of these, 7872 patients remained as the primary study population after application of all exclusion criteria (Figure 1 and Supporting Information S1: Table S1). The most common exclusion criteria were cardiogenic shock and ST‐elevation myocardial infarction (STEMI). Out of the remaining 7872 patients, 2349 (29.8%) underwent PCI with IABP support and 5523 (70.2%) underwent PCI with mAFP support. The characteristics of these patients are displayed in Table 1. Patients receiving mAFP‐support during PCI were older and more likely to be male. Comorbidities such as anemia, diabetes mellitus, and heart failure were more common in patients with mAFP support. Correspondingly, median baseline systolic and diastolic blood pressure and plasma glucose were higher in the mAFP group, whereas median baseline left ventricular ejection fraction and hemoglobin were lower.

**Table 1:** Characteristics of PCI patients with IABP or mAFP support prior to matching. Multiple demographics and comorbidities were different between the study groups prior to matching.

Variables at admission	IABP, *n* (%)	mAFP, *n* (%)	*p* value
*n*	2349	5523	
Age in years, median (IQR)	70.97 [62.43, 79.18]	74.93 [66.63, 81.62]	< 0.001
Age category, *n* (%)			< 0.001
Age (> 75)	892 (38.0)	2750 (49.8)	
Age (≤ 75)	1457 (62.0)	2773 (50.2)	
Sex, *n* (%)			< 0.001
Female	829 (35.3)	1698 (30.7)	
Male	1520 (64.7)	3825 (69.3)	
eGFR category, *n* (%)			< 0.001
eGFR (≥ 60)	1329 (56.6)	1815 (32.9)	
eGFR (30–59)	703 (29.9)	2852 (51.6)	
eGFR (< 30)	317 (13.5)	856 (15.5)	
History of transfusion due to bleeding, *n* (%)	39 (1.7)	168 (3.0)	0.001
History of chronic heart failure, *n* (%)	514 (21.9)	1934 (35.0)	< 0.001
History of anemia, *n* (%)	343 (14.6)	1045 (18.9)	< 0.001
History of insulin‐dependent diabetes, *n* (%)	316 (13.5)	855 (15.5)	0.02
History of liver disease, *n* (%)	109 (4.6)	316 (5.7)	0.06
History of cancer, *n* (%)	319 (13.6)	974 (17.6)	< 0.001
History of HFrEF, *n* (%)	397 (16.9)	1644 (29.8)	< 0.001
eGFR (mL/min/1.73 m^2^), median (IQR)	65.17 [43.61, 87.50]	61.46 [41.73, 83.09]	< 0.001
Creatinine (mg/dL), median (IQR)	1.30 [1.03, 1.70]	1.30 [1.05, 1.70]	0.06
Hemoglobin (g/dL), median (IQR)	12.28 [10.50, 14.00]	11.60 [9.95, 13.24]	< 0.001
Glucose (mg/dL), median (IQR)	139.00 [110.00, 187.50]	124.00 [103.00, 164.00]	< 0.001
Systolic BP (mmHg), median (IQR)*	120.50 [108.50, 134.00]	123.00 [111.00, 136.00]	< 0.001
Diastolic BP (mmHg), median (IQR)*	66.00 [59.00, 73.00]	68.50 [62.00, 77.00]	< 0.001
Mean arterial pressure (mmHg), median (IQR)*	84.00 [76.67, 92.92]	87.00 [79.33, 95.33]	0.08
LVEF (%), median (IQR)*	40.00 [30.00, 55.00]	35.00 [24.15, 50.00]	< 0.001
Number of vessels treated			< 0.001
1	1214 (51.7)	1712 (31.0)	
2	405 (17.2)	1520 (27.5)	
3	142 (6.0)	835 (15.1)	
4+	36 (1.5)	221 (4.0)	
Unknown	552 (23.5)	1235 (22.4)	

*Note:* Data are reported as median with interquartile range (IQR) or number with percentage of the total cohort. *Available in below 50% of the total cohort.

Abbreviation: HFrEF, heart failure with reduced ejection fraction (40% or less).

**Figure 2:**
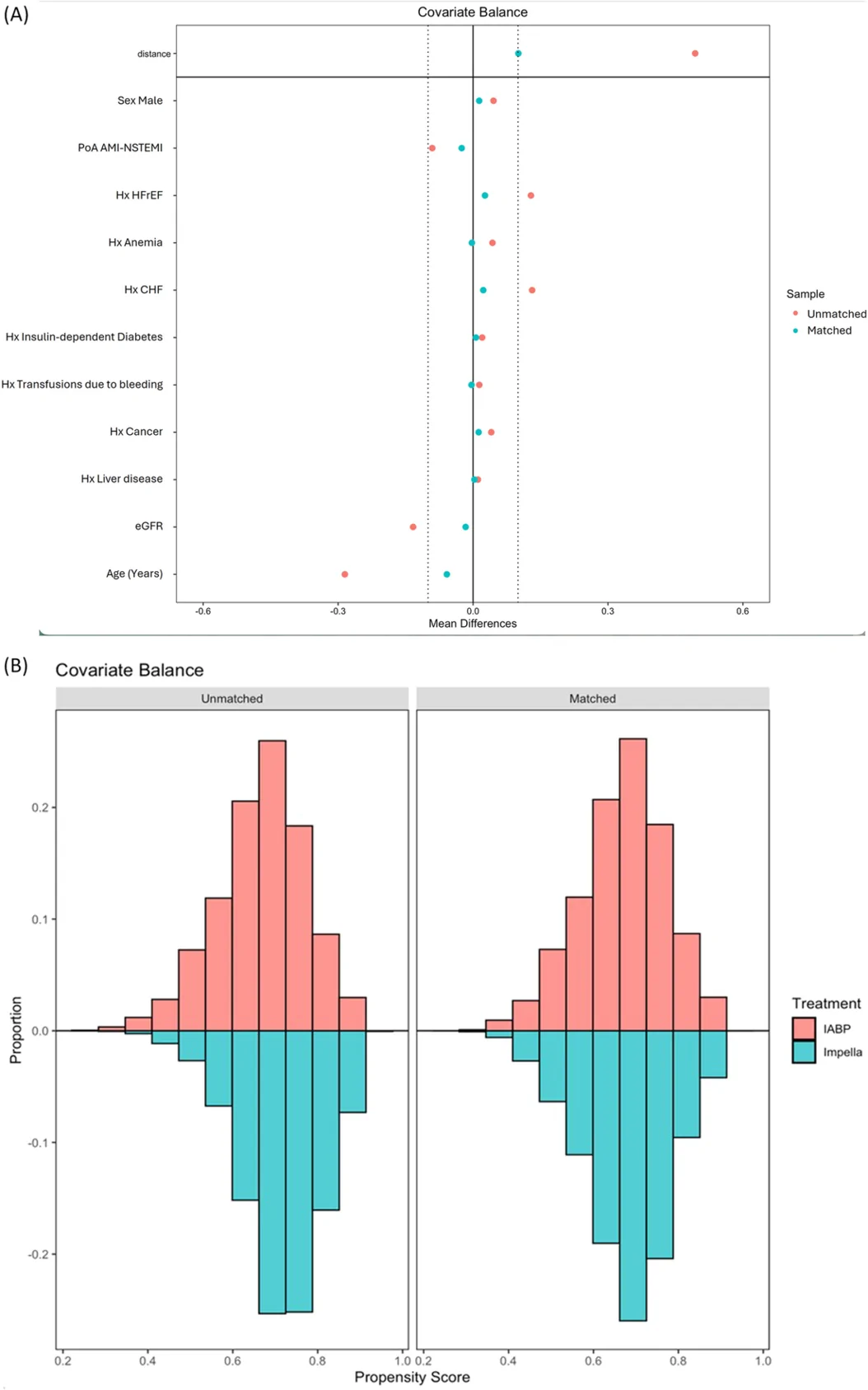
Assessment of covariate balance before and after propensity score matching. (A) Plot of absolute standardized mean differences (SMDs) for each covariate before (red) and after matching (blue). (B) Propensity score distributions for Impella and IABP patients before and after matching. CHF, congestive heart failure; eGFR, estimated glomerular filtration rate; HFrEF, heart failure reduced ejection fraction; Hx, history of; PoA AMI NSTEMI, present on admission acute myocardial infarction non‐ST segment elevation. [Color figure can be viewed at wileyonlinelibrary.com]

Using the prespecified parameters for propensity score matching, including predictors of the risk of AKI, 4666 (2333 per group) patients were matched by the propensity score to receive either support device. The covariate balance for the entire cohort shows that adjusting for AKI risk brought all covariates into the targeted mean difference range (Figure 2A), and covariate balance between treatment groups shows the matched cohort has balanced representation at each propensity score (Figure 2B). Median age of the matched patients was approximately 71 years and two thirds of patients in both groups were male (Table 2). Importantly, there were no differences with respect to demographic parameters, clinical characteristics, or comorbidities between the two propensity score‐matched groups (Table 2). Small, numerical, between‐group differences persisted after matching with respect to some hemodynamic and laboratory parameters (systolic blood pressure and glucose, Table 2), but these were considered not clinically meaningful differences.

**Table 2:** Characteristics of PCI patients with IABP of mAFP support after propensity score matching. Demographics and comorbidities were not different after propensity score matching.

Variables at admission	IABP, *n* (%)	mAFP, *n* (%)	*p* value
*n*	2333	2333	
Age in years, median (IQR)	71.13 [62.63, 79.29]	72.15 [62.88, 80.12]	0.91
Age category, *n* (%)			0.85
Age (> 75)	892 (38.2)	956 (41.0)	
Age (≤ 75)	1441 (61.8)	1377 (59.0)	
Sex, *n* (%)			0.40
Male	1513 (64.9)	1530 (65.6)	
Female	820 (35.1)	803 (34.4)	
eGFR category, *n* (%)			0.76
eGFR (≥ 60)	703 (30.1)	703 (30.1)	
eGFR (30–59)	1313 (56.3)	1296 (55.6)	
eGFR (< 30)	317 (13.6)	334 (14.3)	
Acute myocardial infarction at admission, *n* (%)	1152 (49.4)	1117 (47.9)	0.76
History of transfusion due to bleeding, *n* (%)	39 (1.7)	49 (2.1)	0.85
History of CHF, *n* (%)	514 (22.0)	577 (24.7)	0.43
History of anemia, *n* (%)	342 (14.7)	378 (16.2)	0.494
History of insulin‐dependent diabetes, *n* (%)	314 (13.5)	326 (14.0)	0.16
History of liver disease, *n* (%)	109 (4.7)	111 (4.8)	0.33
History of cancer, *n* (%)	318 (13.6)	361 (15.5)	0.14
History of HFrEF, *n* (%)	397 (17.0)	456 (19.5)	0.26
eGFR (mL/min/1.73 m^2^), median (IQR)	64.93 [43.48, 87.23]	64.48 [43.78, 85.86]	0.06
Creatinine (mg/dL), median (IQR)	1.10 [0.88, 1.50]	1.10 [0.88, 1.50]	0.93
Hemoglobin (g/dL), median (IQR))	12.25 [10.49, 14.00]	11.70 [10.00, 13.30]	0.20
Glucose (mg/dL), median (IQR)	139.00 [110.00, 188.00]	126.00 [103.25, 167.00]	0.04
SBP (mmHg), median (IQR)	120.00 [108.50, 134.00]	123.00 [111.50, 136.00]	0.06
DBP (mmHg), median (IQR)	66.00 [59.00, 73.00]	69.00 [62.00, 77.00]	0.78
MAP (mmHg), median (IQR)	84.00 [76.50, 92.71]	87.33 [79.50, 96.33]	0.80
LVEF (%), median (IQR)	40.00 [30.00, 55.00]	35.00 [25.00, 50.48]	0.07
Number of vessels treated			< 0.001
1	1208 (51.7)	722 (30.9)	
2	404 (17.3)	634 (27.1)	
3	141 (6.0)	338 (14.4)	
4+	36 (1.5)	103 (4.4)	
Unknown	551 (23.6)	543 (23.2)	

*Note:* Data are reported as median with interquartile range (IQR) or number with percentage of the total cohort.

Abbreviations: BP, blood pressure; HFrEF, heart failure with reduced ejection fraction (40% or less); LVEF, left ventricular ejection fraction.

### 3.2 Outcomes in Propensity Score Matched Patients With mAFP Versus IABP Support

**Figure 3:**
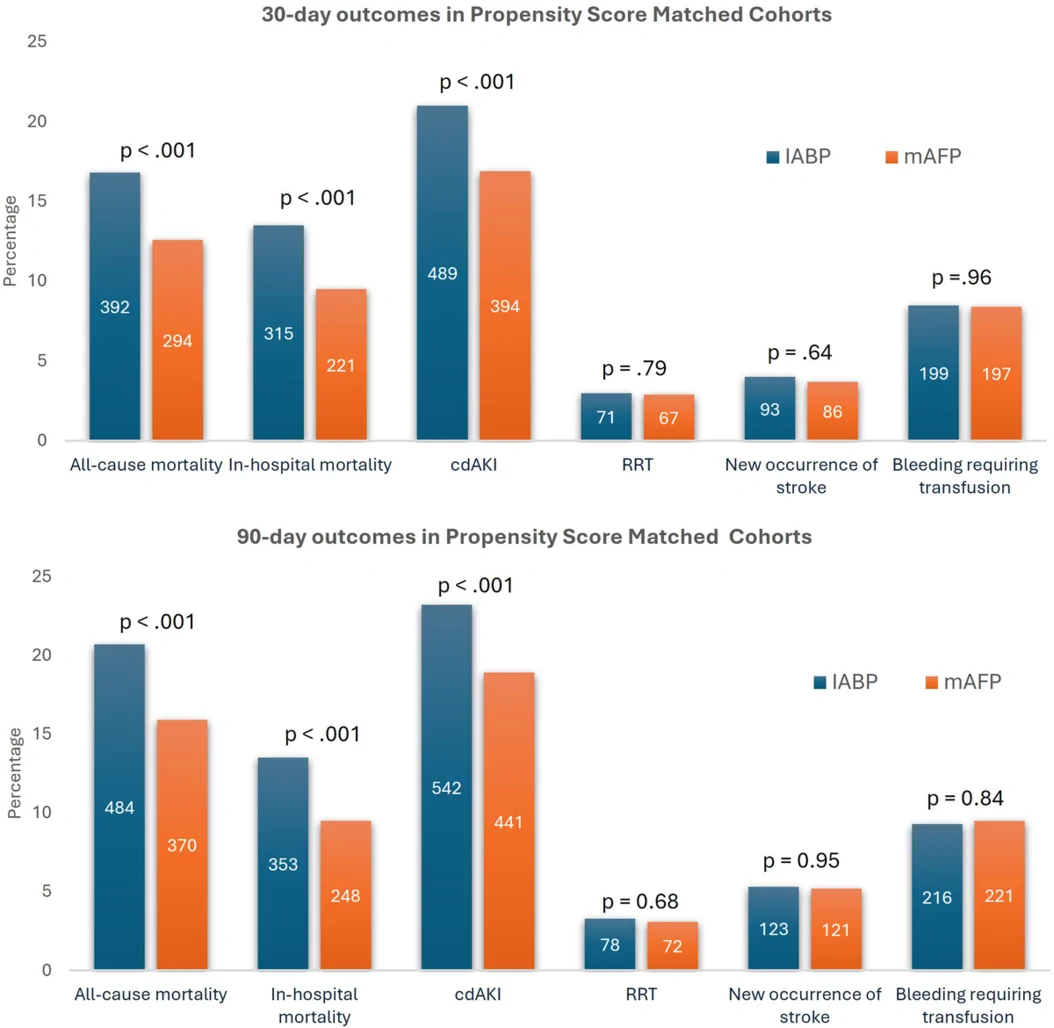
30‐day and 90‐day outcomes in propensity score matched patients with mAFP versus IABP support. Mortality and code‐derived AKI (cdAKI) were lower in the mAFP group compared to IABP at 30 and 90 days. There were no differences in RRT, stroke, or bleeding between groups at any time point. [Color figure can be viewed at wileyonlinelibrary.com]

All outcomes at 30 and 90 days after PCI in the propensity score‐matched cohorts (total *n* = 2333 per group) are displayed in Figure 3.

Through 30 days, cumulatively recorded cd‐AKI, and all‐cause mortality, were higher in patients who underwent PCI with IABP support as compared to those who underwent mAFP support. The risk of AKI was 20% lower in the mAFP compared to the IABP group, while the risk of RRT was not different between groups (3% IABP vs. 2.9% mAFP, *p* = 0.79), likely driven by the relatively low overall use of RRT in the study cohort (3%). The risk of all‐cause mortality was 25% lower at 30 days in mAFP‐supported patients. There were no differences in the new occurrence of stroke or bleeding requiring transfusion between the groups.

Similarly, through 90 days, cumulatively recorded cd‐AKI was 19% lower while the incidence of RRT was not different between groups (3.3% IABP vs. 3.1% mAFP, *p* = 0.68) and all‐cause mortality was ~23% lower in patients with mAFP‐support. Again, rates of stroke or bleeding requiring transfusion did not differ between the groups. Given the higher survival in the mAFP group, to address potential survivorship bias, we performed an analysis of renal events among 30‐ and 90‐day survivors. Among survivors, the incidence of AKI was still significantly lower in the mAFP group compared to the IABP group (Supporting Information S1: Tables S2, S3). Given the long temporal span (> 8 years) in which the data was collected, a subgroup analysis was conducted to assess early versus late treatment‐related outcomes. Crude differences in mortality between Impella and IABP patients were consistent over this time period (see Supporting Information S1: Table S9).

We proceeded to test whether the association of the device choice and AKI was subject to interaction with the baseline kidney function using saturated logistic regression models with an interaction term. Here, CKD stages IIIa−V were associated with higher risk compared to CKD stages I−II, but we did not detect evidence of statistically significant interaction between baseline eGFR category and device choice with respect to AKI incidence. While there was no statistically significant interaction, the greatest apparent cd‐AKI risk difference in favor of mAFP versus IABP was observed in CKD stages I−IIIa (Table 3).

**Table 3:** Risk of AKI according to baseline kidney function in the matched cohort. Comparison of AKI rates between Impella and IABP across the four CKD stages showed no significant association between baseline CKD level, treatment group, and AKI rates (Cochran−Mantel−Haenszel test *p* = 0.1151).

CKD stage	eGFR	IABP (*N* = 2333)	mAFP (*N* = 2333)	
		***N* (%)**	***N* (%)**	**Crude odds ratio (mAFP vs. IABP)**
CKD I and II	≥ 60	220/1334 (16)	170/1331 (13)	0.74 (0.59–0.93)
CKD IIIa	45−59	129/376 (34)	99/389 (25)	0.65 (0.47–0.90)
CKD IIIb	30−44	101/319 (32)	89/289 (31)	0.96 (0.67–1.37)
CKD IV/V	≤ 29	92/304 (30)	83/324 (26)	0.79 (0.55–1.14)

*Note:* Crude odds ratios were calculated with chi‐square tests within each CKD category without correction for multiple testing.

Abbreviation: CKD, chronic kidney disease stage.

To identify the clinical implications of the renal endpoints that were tracked in this analysis, we analyzed whether the incidence of AKI or the initiation of RRT were associated with higher mortality at 90‐days post‐PCI. Indeed, all these renal events were associated with higher 90‐day mortality across both treatment groups, as well as within each treatment group (Table 4).

**Table 4:** Association of renal endpoints with 90‐day all‐cause mortality across and within un‐matched and matched groups. AKI and RRT were associated with higher mortality in both groups.

	**Unmatched cohort**			**Matched cohort**		
	Both (OR [95% CI])	IABP (*N* = 2349) (OR [95% CI])	mAFP (*N* = 5523) (OR [95% CI])	Both (OR [95% CI])	IABP (*N* = 2333) (OR [95% CI])	mAFP (*N* = 2333) (OR [95% CI])
All‐cause mortality with AKI Number of deaths/numbers of AKI	1.75 (1.53−2.00) 410/1709	1.49 (1.19−1.87) 141/543	1.89 (1.60−2.22) 269/1166	1.59 (1.34−1.88) 238/983	1.48 (1.18−1.85) 141/542	1.67 (1.28−2.16) 97/441
All‐cause mortality with RRT Number of deaths/numbers of RRT	4.10 (3.16−5.31) 110/248	3.88 (2.45−6.13) 38/78	4.22 (3.07−5.77) 72/170	3.31 (2.36−4.61) 62/150	3.85 (2.43−6.08) 38/72	2.76 (1.64−4.53) 24/72

*Note:* In each cell, in the top line, the odds ratio of all‐cause mortality is displayed for those patients who experienced the respective renal event as compared to those who did not experience this event. The odds ratios are presented across both groups (first column) or within the groups (second and third column).

Abbreviations: AKI, acute kidney injury; RRT, renal replacement therapy.

## 4 Discussion

We here demonstrate in a large RWD set that the use of mAFP during high‑risk PCI is associated with a lower risk of AKI and mortality compared to IABP in a propensity score‐matched sample.

Interestingly, the above‐mentioned differences in mortality and renal outcomes were also present in this study before matching, even though, in the unmatched sample, mAFP‐supported patients were older, more likely male, had higher systolic and diastolic blood pressure, and had more comorbidities than patients undergoing high‐risk PCI with IABP support. Compared with the PROTECT II [15] randomized controlled trial as well as contemporary registries [16], patients revascularized with mAFP support in the present study were older. It is relevant to note that this is also a display of varying patient selection for these devices in US clinical practice, which we approached using propensity score matching. Our results demonstrate the feasibility of applying propensity score matching in a large database like TRUVETA for such confounders. This correction should allow for a meaningful comparison between treatment groups, even though residual confounding is likely and the results remain hypothesis‐generating [14].

While both the mAFP and the IABP aim to stabilize hemodynamics, their mechanisms and hemodynamic effects differ, particularly with respect to effects on cardiac filling pressures and volumes and downstream end‐organ perfusion [17]. In short, the mAFP offers continuous left ventricular unloading, directly increasing end‐organ (i.e., renal) perfusion [18], whereas the effects of counterpulsation devices like the IABP on distally located renal perfusion are unknown. Further mechanistic, comparative studies are required in this context. Another potential reason for the observed differences in AKI and mortality rates between mAFP and IABP may be due to mAFP‐driven reduction in left ventricular pressures and volumes. This may impact the cardiorenal axis via reducing excretion of natriuretic peptides and the decrease of cardiac sympathetic nerve outflow, leading to inhibition of the renin‐angiotensin‐aldosterone system (RAAS).

Previous preclinical and observational data has hinted toward potential nephroprotective effects of mAFPs. In a single‐center study, lower incidences of AKI in high‐risk PCI patients supported with mAFP compared to ECMO were reported [19]. A retrospective study by Flaherty et al. matched 115 mAFP (Impella 2.5)‐supported patients undergoing high‐risk PCI to non‐supported patients undergoing high‐risk PCI and identified a significantly reduced risk of AKI and RRT [7]. Furthermore, a prospective, multicenter observational study that enrolled 223 patients undergoing Impella 2.5 or CP‐supported high‐risk PCI, rates of AKI were lower than expected based on existing risk scores, again suggesting a renal benefit of these devices [6].

However, these high‐risk PCI data have recently been challenged by the results of the DanGer Shock trial which demonstrated not only lower 180‐day mortality rates in patients with infarct‐related cardiogenic shock who received mAFP support but also increased rates of AKI and RRT [8, 9]. Collectively, these data suggest that the renal effects of mAFP differ significantly between high‐risk PCI and cardiogenic shock. In the DanGer trial, hypoperfusion and profound metabolic abnormalities in shock precede the use of mAFP in the DanGer study by an average of 4.8 (2.4−12.8) h. It could therefore be speculated that this difference in renal risk with mAFP use between high‐risk PCI and cardiogenic shock arises from the hemodynamic compromise (i.e., renal hypoperfusion) and increased venous congestion in cardiogenic shock that increases renal vulnerability to further stressors, whereas mAFP‐supported high‐risk PCI patients do not typically experience such severe hemodynamic fluctuations. In turn, an additional stressor such as device‐related hemolysis may represent a more significant threat to the kidney in the setting of cardiogenic shock than in high‐risk PCI. Accordingly, in DanGer Shock, the major risk factors for AKI in shock that were specific to the mAFP group included high pump speeds, suction events, and longer duration of support [8]. The trial protocol required that the mAFP be operated at the highest possible power level for at least 24 h. Such continuous high pump speeds and long duration of support are not typically seen in high‐risk PCI but will inevitably increase the burden of hemolysis. Even though such considerations comparing the present study's result to DanGer Shock remain speculative due to the drastically different target populations, they may aid in the conceptualization of future research to address these outcomes.

The present study only compared the utilization of outcomes between the two devices without a no device control group, because residual confounding would have likely been substantial. Thus, it is not possible to estimate in this sample what the incidence of AKI would have been without the utilization of either of these devices. In this context, the effects of IABP use on renal outcomes—both in cardiogenic shock and high‐risk PCI—remain unknown. In the IABP‐SHOCK II trial, allocation to IABP had no effect on early renal function but was associated with a 31% higher rate of RRT [20]. In addition, a retrospective study by Tsai et al. identified IABP as a risk factor for AKI after PCI [21].

### 4.1 Limitations

The present study leverages the largest existing RWD set with laboratory parameters to examine renal outcomes and demonstrates the feasibility of such an analysis. However, it is important to note that the renal outcomes in the present analysis, that is, AKI and RRT, occurred relatively infrequently, when compared with previous reports which estimated the rate of AKI after high‐risk PCI (without MCS) to be approximately 28% [7]. This is likely related to the assessment of these outcomes and to the specific inclusion and exclusion criteria applied to this analysis, for example, the exclusion of patients with cardiogenic shock or emergent PCI.

Of course, these and other limitations arise from the nature of the data source. Correspondingly, other variables, such as longitudinal serum creatinine post‐PCI, that would have been of interest for calculation of longitudinal eGFR and AKI events, could not be included due to highly limited or no data availability. Instead, AKI events were investigated using a code derived definition. However, code derived AKI is limited by delays in administrative logging and therefore has poor temporal resolution limiting conclusions to cumulative incidence of events over large time periods. The timing of MCS and the use case under which MCS was used were not fully controlled for in this study due to a lack of necessary data in the RWD set. Furthermore, important baseline variables such as lactate, cardiac enzymes, and invasive right heart catheterization hemodynamics had high missingness in the data set (> 80% missing) and were not able to be used to characterize the patient or perform propensity matching. Despite including only elective patients, and excluding cardiogenic shock patients, which likely narrowed the use case and timing of MCS, residual confounders from MCS timing may be present. Finally, although propensity score matching was used in this study to allow causal inference and to limit potential confounding by covariates, residual confounding, potentially in the form of indication bias from the use of administrative data [22], is likely and causal interpretations of the reported data cannot be made. Further, residual confounding reducing methods such as a falsification negative control endpoint not expected to change between groups (e.g., pneumonia outcome) may further help assess the presence of confounders not fully addressed with propensity matching but is outside the scope of this manuscript.

However, the major strengths of the present approach lie in the exceptionally large study population and the use of RWD outside the barriers and selection biases to which clinical trials are usually prone. Because of the design of the Truveta database, it is reasonable to assume that the results of this analysis are reflective of the US population, even though a subset of patients had to be excluded due to missing data (eGFR), which may limit generalizability [23].

## 5 Conclusion

This propensity score‐matched observational study provides real‐world evidence that the use of mAFP in the setting of elective high‐risk PCI is associated with a lower risk of AKI and improved 90 days survival compared to the use of an IABP. Due to the retrospective nature of the study, despite propensity score matching, residual confounding is likely, and further investigations are needed to evaluate the mechanisms of renal protection or injury with these MCS devices. This research provides a rationale for further investigations to evaluate the mechanisms of renal protection or injury with these MCS devices (Central Illustration 1).

**Central Illustration 1:**
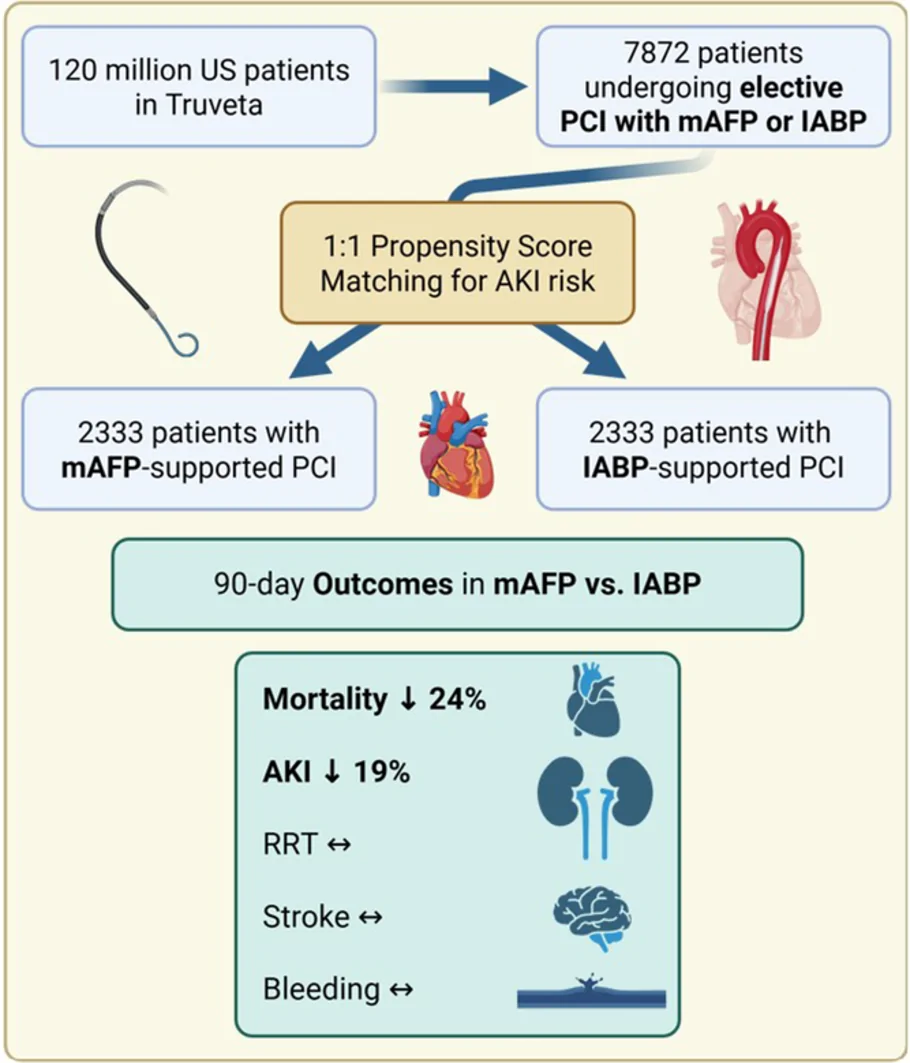
AKI, acute kidney injury; IABP, intra‐aortic balloon pump; mAFP, microaxial flow pump; MAKE, major adverse kidney events (composite of mortality and renal replacement therapy); PCI, percutaneous coronary intervention; RRT, renal replacement therapy. Generated with BioRender. Microaxial flow pump support during elective PCI is associated with lower AKI risk and higher survival compared to intra‐aotric balloon pump support in a propensity score‐matched real‐world sample. [Color figure can be viewed at wileyonlinelibrary.com]

## Conflicts of Interest

E.Z. reports travel grants and speaker honoraria from Abiomed. A.P. reports institutional research grants and personal speakers' honoraria from Abiomed Inc. R.K. is a founder, shareholder, and board member of Sequantrix GmbH; is a member of the scientific advisory board of Hybridize Therapeutics; has received honoraria for advisory boards and talks from Bayer, Chugai, Pfizer, Roche, Genentech, Eli Lilly, AMGEN, Novo Nordisk, and GlaxoSmithKline, Abiomed; and has research funding from Chugai, Travere Therapeutics, Galapagos, Novo Nordisk, and AskBio. N.N., S.U., M.C., and R.W. are employed by Abiomed.

## Supporting information

Supporting File

## Data Availability

The data used for this manuscript are obtained from the TRUVETA data set, which is commercially available at www.truveta.com and can therefore not be shared publicly.
