# Supplementary material for: Lower AKI Risk and Higher Survival With Microaxial Flow Pump Support During Elective PCI: A Propensity Score‐Matched Study
Source: Catheter Cardiovasc Interv. 2026 Jul 6;108(3):935–45. doi: 10.1002/ccd.70721 (PMC13532450; doi:10.1002/ccd.70721)
Supplement: Supplementary file 1 [file CCD-108-935-s001.docx]

# Lower AKI Risk and Higher Survival with Microaxial Flow Pump Support During Elective PCI: A Propensity Score-Matched Study

Elric Zweck, MD^1^, Andrew Shaw, MD^2^, Amin Polzin, MD^1^, Nudrat Noor, PhD^2^, Sathya Unudurthi, PhD, MBA^2^, Michael Cuchiara, PhD^2^, Kai M. Schmidt-Ott, MD, PhD^3^, Malte Kelm, MD^1^, Ralf Westenfeld, MD^1,2^, Rafael Kramann, MD, PhD^4,5#^, Ravindra L. Mehta, MD^6#^

^1^ Department of Cardiology, Pulmonology, and Vascular Medicine, Medical Faculty, Heinrich Heine University Düsseldorf, Düsseldorf, Germany

^2^ Abiomed, Johnson & Johnson MedTech, Danvers, MA, USA

^3^ Department of Nephrology and Hypertension, Hannover Medical School, Hannover, Germany

^4^ Department of Medicine 2 (Nephrology, Rheumatology, Immunology), Medical Faculty, RWTH Aachen University, Aachen, Germany

^5^ Department of Internal Medicine, Nephrology and Transplantation, Erasmus Medical Center, Rotterdam, The Netherlands

^6^ Department of Medicine, University of California San Diego, CA, USA

^#^joint corresponding authorship.

*Correspondence to:

Prof. Dr. Rafael Kramann

Department of Medicine 2 (Nephrology, Rheumatology, Immunology)

Medical Faculty, RWTH Aachen University

Pauwelsstraße 30

52074 Aachen

Germany

Tel.: +49 241 80-37750

Mail: rkramann@ukaachen.de

Access to the database for this study was funded by Johnson & Johnson MedTech HeartRecovery.

**Supplemental Table S1: Patient Eligibility**

| **Attrition Table** | **TOTAL** | | | **IABP** | | | **mAFP** | | |
| --- | --- | --- | --- | --- | --- | --- | --- | --- | --- |
|  |  |  |  | **Retained** | **Removed** | **%**  **Removed** | **Retained** | **Removed** | **%**  **Removed** |
|  | **Retained** | **Removed** | **%**  **Removed** |  |  |  |  |  |  |
| **ALL PCI's** (01/01/2017 - 08/31/2024) | 847,985 |  | - | - | - | - | - | - | - |
| **Remove patients with no PCI-Date** | 615,885 | 232,100 | 27.4% |  | - | - | - | - | - |
| Restrict to most recent MCS-supported PCI ^a^ | 24,645 | 591,240 | 96.0% | 11,564 | 456 | 4% | 14,201 | 615 | 5% |
| Exclude ECMO ^b^ | 23,962 | 683 | 2.8% | 11,381 | 183 | 1.6% | 13,637 | 564 | 4.0% |
| Remove Multiple Devices (multiple devices on PCI-date) | 22,906 | 1,056 | 4.4% | 10,325 | 1,056 | 9.3% | 12,581 | 1,056 | 7.7% |
| Exclude emergent admission | 22,069 | 837 | 3.7% | 9,905 | 420 | 4.1% | 12,164 | 417 | 3.3% |
| Exclude CS ^c^ | 15,500 | 6,569 | 29.8% | 6,768 | 3,137 | 31.7% | 8,732 | 3,432 | 28.2% |
| Exclude AMI/STEMI ^c^ | 10,459 | 5,041 | 32.5% | 3,250 | 3,518 | 52.0% | 7,209 | 1,523 | 17.4% |
| Exclude RHF ^c^ | 10,391 | 68 | 0.7% | 3,229 | 21 | 0.6% | 7,162 | 47 | 0.7% |
| Exclude CABG ^c^ | 10,147 | 244 | 2.3% | 3,038 | 191 | 5.9% | 7,109 | 53 | 0.7% |
| **Remove patients with missing demographics data** | 10,144 | 3 | 0.0% | 3,037 | 1 | 0.0% | 7,107 | 2 | 0.0% |
| **Exclude patients with missing eGFR** | 7,872 | 2,272 | 22.4% | 2,349 | 688 | 22.7% | 5,523 | 1,584 | 22.3% |
| **PROPENSITY MATCHED (1:1)** | **4666** |  |  | **2333** |  |  | **2333** |  |  |

a: Restrict to earliest PCI; does not exclude multiple devices. Earliest device used classified under Impella or IABP. b: remove ECMO usage. c: exclusions is -7 to -0d of admission date.

**Supplemental Table S2: 30-day renal outcomes among 30-day survivors of the propensity score matched patients with mAFP versus IABP support**

| **Variables** | **IABP, n (%)** | **mAFP, n (%)** | **P Value** |
| --- | --- | --- | --- |
| **n** | 617 | 662 |  |
| **AKI ^[1]^ – n (%)** | 379 (19.5) | 318 (15.5) | **.001** |
| **Renal Replacement Therapy – n (%)** | 42 (2.16) | 47 (2.30) | .85 |

^1^New occurrence of kidney failure: any of the following: Diagnosis of N14 (N14.1, N14.11, N14.19). Diagnosis of N17 (including: N17.0, N17.1, N17.2, N17.8, N17.9) acute kidney failure NOT present on admission OR (N19 or N99.0 or T86.12) Diagnosis of kidney failure unspecified NOT present on admission OR (N18.6) Diagnoses of chronic kidney disease end-stage NOT present on admission

**Supplemental Table S3: 90-day renal outcomes among 90-day survivors of the propensity score matched patients with mAFP versus IABP support**

| **Variables** | **IABP, n (%)** | **mAFP, n (%)** | **P Value** |
| --- | --- | --- | --- |
| **n** | 599 | 632 |  |
| **AKI ^[1]^ – n (%)** | 401 (17.5) | 334 (21.6) | **.001** |
| **Renal Replacement Therapy – n (%)** | 40 (2.16) | 48 (2.44) | .64 |

^1^New occurrence of kidney failure: any of the following: Diagnosis of N14 (N14.1, N14.11, N14.19). Diagnosis of N17 (including: N17.0, N17.1, N17.2, N17.8, N17.9) acute kidney failure NOT present on admission OR (N19 or N99.0 or T86.12) Diagnosis of kidney failure unspecified NOT present on admission OR (N18.6) Diagnoses of chronic kidney disease end-stage NOT present on admission

**Supplemental Table S4: Saturated logistic regression models for the risk of AKI by device choice and baseline kidney function including interaction terms**

|  | OR | 2.5%CI | 97.5% CI | p-value |
| --- | --- | --- | --- | --- |
| Impella | 0.72 | 0.58 | 0.90 | .003 |
| CKD IIIa | 2.70 | 2.08 | 3.49 | <.001 |
| CKD IIIb | 2.35 | 1.75 | 3.13 | <.001 |
| CKD IV-V | 2.24 | 1.68 | 2.98 | <.001 |
| Impella*CKD IIIa | 0.91 | 0.62 | 1.33 | .60 |
| Impella*CKD IIIb | 1.28 | 0.83 | 1.96 | .26 |
| Impella*CKD IV-V | 1.10 | 0.73 | 1.66 | .65 |

The displayed model corresponds to the equation of ln(OR) = β_0_ + β_1_*device + β_2_*CKD_stage + β_3_*device*CKD_stage with IABP and CKD stage I-II as the reference groups.

**Supplemental Table S5: 30-day outcomes in propensity score matched patients with mAFP versus IABP support**

|  | **IABP**  **n(%)** | **mAFP**  n(%) | **mAFP vs. IABP [OR (95% CI)]** |
| --- | --- | --- | --- |
| **All-cause Mortality** | 149 (20) | 104 (14) | 0.94 (0.90-0.98) |
| **AKI** | 104 (14) | 72 (9) | 0.95 (0.92-0.99) |

**Supplemental Table S6: 90-day outcomes in propensity score matched patients with mAFP versus IABP support**

|  | **IABP**  **n(%)** | **mAFP**  n(%) | **mAFP vs. IABP [OR (95% CI)]** |
| --- | --- | --- | --- |
| **All-cause Mortality** | 167 (22) | 134 (18) | 0.96 (0.92-0.99) |
| **AKI** | 122 (16) | 86 (11) | 0.95 (0.92-0.99) |

| **Event** | **Codes Used** |
| --- | --- |
| Acute kidney injury | ICD10CM: N14 (including: N14.1, N14.11, N14.19), N17 (including: N17.0, N17.1, N17.2, N17.8, N17.9), N19, N99.0, T86.12, or N18.6. |
| Renal Replacement Therapy | CPT: 0947,90966,90970,90989,90935,90945,3066F,90937,90993,4054F,4055F,90999,,90925,909213E1M39Z  ICD10PCS: 3E1M39Z |
| Stroke | ICD10CM : I63.523 I63.332 I63.89 I63.311 I63.02 I63.349 I63.81 I60.10 I63.019 I63.429 I60.50 I63.4 I60.11 I63.10 I63.20 I63.31 I60.6 I63.53 I63.0 I60.30 I63.031 I63.42 I63.01 I63.00 I61.2 I60.9 I61.6 I63.543 I63.139 I63.119 I63.329 I60.21 I63.212 I61.9 I62.00 I63.449 I63.3 I60.02 I62.02 I60.01 I63.1 I63.313 I63.239 I63.012 I63.442 I63.443 I61.1 I63.521 I63.39 I62.1 I63.132 I63.411 I63.34 I61.5 I63.29 G45.3 G45.8 I63.5 I63.032 I63.433 I63.54 I63.8 I63.322 I60.2 I60.22 I63.339 I63.49 I63.512 I60.5 I63.319 I63.2 I62.03 I63.22 I61.8 I63.9 I63.23 I63.533 I60.3 I60.1 I63.341 I63.43 I63.13 I63.6 I63.321 I63.233 I63.342 I63.219 I61 I63.531 I60.0 I63.542 I63.549 I60.32 I63.12 I63.513 I60.8 I63.532 I63.539 I63.312 I60.4 I63.211 I63.441 I60.52 I63.232 I63.32 I60 I63.323 G45 I63.431 I61.0 I63.40 G45.2 I60.7 I63.412 I63.112 I62.0 I63.343 I63.11 I62 G45.1 I63.111 I63.213 I63.422 I63.133 I63.113 I63.519 I63.30 I63.011 I63.039 I60.20 I63.41 I63.419 I62.9 G45.0 I60.00 I63.09 I63.33 I63.51 I63.21 I63.231 I63.423 I63.19 I60.31 I63.52 I63.522 I63.03 G45.4 I61.4 I63.439 I63.331 I63.511 I63.131 I60.51 I62.01 I63 I63.541 I63.033 I63.59 I63.529 I60.12 I63.44 I63.50 I63.413 I61.3 I63.333 I63.421 I63.432 G45.9 I63.013 |
| Transfusion due to bleeding | ICD10 PCS: 30253P1 30233H0 30263H1 30243N0 30243N1 30253H1 30263P1 30263H0 30233N0 30233H1 30233N1 30253N1 30263N1 30233P1 30243H1 30253N0 30263N0 30253H0 30243H0 30243P  ICD10 CM codes: I97.51 I97.620 I97.621 I97.418 I97.630 I97.52 I97.42 I97.610 I97.638 I97.618 I97.622 I97.62 I97.411 I97.410 I97.631 I97.611 K66.1 |

**Supplemental Table S7: Codes used to define outcomes**

**Supplemental Table S8: Unadjusted outcomes between the mAFP and IABP groups prior to propensity score matching.**

| **Variables** | **IABP, n (%)** | **mAFP, n (%)** | **SMD** | **P Value** |
| --- | --- | --- | --- | --- |
| **n** | 2349 | 5523 |  |  |
| All-cause mortality (30 day) | 392 (16.7) | 651 (11.8) | .14 | <.001 |
| All-cause mortality (90 day) | 484 (20.6) | 865 (15.7) | .13 | <.001 |
| In-hospital mortality (30 day) | 315 (13.4) | 492 ( 8.9) | .14 | <.001 |
| In-hospital mortality (90 day) | 353 (15.0) | 559 (10.1) | .15 | <.001 |
| Renal Replacement Therapy (30d) | 71 ( 3.0) | 159 ( 2.9) | .01 | .48 |
| Renal Replacement Therapy (90d) | 78 ( 3.3) | 170 ( 3.1) | .01 | .70 |
| Acute Kidney Injury (30 day) | 489 (20.8) | 1030 (18.6) | .05 | .02 |
| Acute Kidney Injury (90 day) | 543 (23.1) | 1166 (21.1) | .05 | .052 |
| Stroke (30 day) | 93 ( 4.0) | 211 ( 3.8) | .01 | .98 |
| Stroke (90 day) | 123 ( 5.2) | 292 ( 5.3) | .002 | .78 |
| Bleeding requiring transfusion (30 day) | 179 ( 7.6) | 413 ( 7.5) | .01 | .86 |
| Bleeding requiring transfusion (90 day) | 195 ( 8.3) | 456 ( 8.3) | .002 | .98 |

**Supplemental Table S9: Sensitivity analysis stratifying by era (e.g., early vs. late study period).**

|  | **Early period^1^** | | | **Late period^1^** | | |
| --- | --- | --- | --- | --- | --- | --- |
| **Variables** | **IABP** | **mAFP** | **P Value** | **IABP** | **mAFP** | **P Value** |
| **n** | 910 | 1533 |  | 1439 | 3990 |  |
|  | **n (%)** | **n (%)** |  | **n (%)** | **n (%)** |  |
| All-cause mortality (30 day) | 161 (17.7) | 177 (11.5) | <.001 | 231 (16.1) | 474 (11.9) | <.001 |
| All-cause mortality (90 day) | 196 (21.5) | 237 (15.5) | <.001 | 288 (20.0) | 628 (15.7) | <.001 |
| In-hospital mortality (30 day) | 132 (14.5) | 151 ( 9.8) | <.001 | 183 (12.7) | 341 ( 8.5) | <.001 |
| In-hospital mortality (90 day) | 151 (16.6) | 169 (11.0) | <.001 | 202 (14.0) | 390 ( 9.8) | <.001 |
| Renal Replacement Therapy (30d) | 32 ( 3.5) | 34 ( 2.2) | .07 | 39 ( 2.7) | 125 ( 3.1) | .48 |
| Renal Replacement Therapy (90d) | 33 ( 3.6) | 35 ( 2.3) | .07 | 45 ( 3.1) | 135 ( 3.4) | .70 |
| Acute Kidney Injury (30 day) | 176 (19.3) | 279 (18.2) | .52 | 313 (21.8) | 751 (18.8) | .02 |
| Acute Kidney Injury (90 day) | 199 (21.9) | 313 (20.4) | .42 | 344 (23.9) | 853 (21.4) | .052 |
| Stroke (30 day) | 38 ( 4.2) | 56 ( 3.7) | .59 | 55 ( 3.8) | 155 ( 3.9) | .98 |
| Stroke (90 day) | 51 ( 5.6) | 83 ( 5.4) | .91 | 72 ( 5.0) | 209 ( 5.2) | .78 |
| Bleeding requiring transfusion (30 day) | 70 ( 7.7) | 113 ( 7.4) | .83 | 109 ( 7.6) | 300 ( 7.5) | .99 |
| Bleeding requiring transfusion (90 day) | 74 ( 8.1) | 124 ( 8.1) | .999 | 121 ( 8.4) | 332 ( 8.3) | .96 |

**^1^** "Early period" is defined as receiving device in the period 2020 or earlier, and "Late period" is defined as receiving device in the period 2021-2025.
